# Understanding variability in optimum plant density and recommendation domains for crowding stress tolerant processing sweet corn

**DOI:** 10.1371/journal.pone.0228809

**Published:** 2020-02-07

**Authors:** Daljeet S. Dhaliwal, Martin M. Williams

**Affiliations:** 1 Department of Crop Sciences, University of Illinois, Urbana, Illinois, United States of America; 2 Global Change and Photosynthesis Research Unit, USDA-ARS, Urbana, Illinois, United States of America; Harran University, TURKEY

## Abstract

Recent research shows significant economic benefit if the processing sweet corn [*Zea mays* L. var. *rugosa* (or *saccharata*)] industry grew crowding stress tolerant (CST) hybrids at their optimum plant densities, which may exceed current plant densities by up to 14,500 plants ha^-1^. However, optimum plant density of individual fields varies over years and across the Upper Midwest (Illinois, Minnesota and Wisconsin), where processing sweet corn is concentrated. The objectives of this study were to: (1) determine the extent to which environmental and management practices affect optimum plant density and, (2) identify the most appropriate recommendation domain for making decisions on plant density. To capture spatial and temporal variability in optimum plant density, on-farm experiments were conducted at thirty fields across the states of Illinois, Minnesota and Wisconsin, from 2013 to 2017. Exploratory factor analysis of twelve environmental and management variables revealed two factors, one related to growing period and the other defining soil type, which explained the maximum variability observed across all the fields. These factors were then used to quantify the strength of associations with optimum plant density. Pearson’s partial correlation coefficients of ‘growing period’ and ‘soil type’ with optimum plant density were low (*ρ*_*1*_ = -0.14 and *ρ*_*2*_ = -0.09, respectively) and non-significant (P = 0.47 and 0.65, respectively). To address the second objective, six candidate recommendation domain models (RDM) were developed and tested. Linear mixed effects models describing crop response to plant density were fit to each level of each candidate RDM. The difference in profitability observed at the current plant density for a field and the optimum plant density under RDM level represented the additional processor profit ($ ha^-1^) from a field. The RDM built around ‘Production Area’ (RDM_PA_) appears most suitable, because plant density recommendations based on RDM_PA_ maximized processor profits as well grower returns better than other RDMs. Compared to current plant density, processor profits and grower returns increased by $448 ha^-1^ and $82 ha^-1^, respectively at plant densities under RDM_PA_.

## Introduction

Optimum plant density is essential to maximizing yield in field corn (*Zea mays* L.) [1,2]. Plant density affects plant architecture, alters growth and developmental patterns, and influences carbohydrate production and partitioning [3]. Plant density interactions with environment and crop management practices also can affect crop performance. Shanahan et al. [4] demonstrated field-scale management of plant density as an economically feasible option for field corn production in the western U.S. Corn Belt.

Geographic location and environmental factors such as temperature, precipitation and radiation influence plant density decisions. Assefa et al. [5] reported that as latitude increased from 30°N to 50°N, higher plant densities were required to attain the same yield level as at lower latitudes. At similar plant densities, lower yield in field corn at higher latitudes can be due to decreased amount of solar radiation and reduced crop growing season [6,7]. In southern climates, Thompson et al. [8] found that higher nighttime temperatures were unfavorable for field corn yields and reduced crop yield in above-average plant densities.

Water supply is essential in decision-making for plant density in sweet corn. Compared to irrigated production systems, lower plant densities are recommended for rainfed production. For instance, sweet corn plant densities recommended for irrigated production systems in Minnesota average 66,000 plants ha^-1^, while 55,000 plants ha^-1^ are recommended for rainfed production systems [9]. Higher plant densities can be detrimental for field corn yields during periods of extended water shortage in rainfed production systems [10–12]. When drought is a threat, Norwood [10] suggested hybrid maturity and planting date should be considered when making decisions on plant density.

Previous studies have reported that widely used processing sweet corn hybrids differ greatly in crowding stress tolerance (CST) and yield potential [13,14]. Williams [13] reported that processing sweet corn germplasm with improved CST was under-planted by growers in the Upper Midwest. Dhaliwal and Williams [15] quantified optimum plant density for CST processing sweet corn in the same region. The study reported that CST sweet corn is under-planted 14,500 plants ha^-1^ averaged across thirty fields in the region. Using optimum plant density for CST sweet corn, vegetable processors may realize up to $700 ha^-1^ additional profits [15]. However, optimum plant density varied across space and time. Conceivably, making recommendations for plant density of CST sweet corn tailored to address field-scale variability may increase profitability of both growers and vegetable processors. Vegetable processor profitability is measured as gross profit margin ($ ha^-1^), which in this instance is the value of cases of sweet corn produced per hectare less the contract price paid to the grower and seed costs, measured in $ ha^-1^. Each case contained 6.13 kg of kernels, moisture-corrected at 76 percent. Grower returns ($ ha^-1^) depend on the total green ear mass of sweet corn harvested by processor.

A recommendation domain is defined as “a group of roughly homogeneous farmers with similar circumstances for whom we can make more or less the same recommendation” [16]. Natural circumstances (e.g. biotic factors, climate, soil) and socio-economic factors (e.g. farm size, labor accessibility, power source) are commonly used factors in forming recommendation domains [17]. For instance, two recommendation domains for farming a region of South American highlands were identified; specifically, flat lands and steep lands [18]. Major differences in the methods of land preparation, choice of cultivars and weed management practices were reported between recommendation domains.

Previous studies have reported that targeting sites under the same recommendation domain with the new technology, and for which the technology is suitable, increases the likelihood of adoption of new technology [19,20]. Recommendation domains prevent extrapolating results from better environments to poorer environments [21]. Furthermore, appropriate recommendation domains can avoid two equally undesirable situations of (a) offering a different recommendation when unnecessary, which adds cost, or (b) offering a single recommendation when multiple recommendations are needed [17]. Moreover, effective recommendation domains can guide policy makers in allocating resources appropriately [17].

The goal of this work was to determine the best approach for making plant density recommendations that would maximize the economic benefit of increasing plant densities of CST sweet corn. A previous study with fresh market sweet corn from Connecticut reported gross returns increased by $1,150 ha^-1^ on increasing the plant density from 65,340 to 104,550 plants ha^-1^ [22]. Stanger and Lauer [23] reported variation in optimum plant density for field corn in the Upper Midwest based on local soil and climatic conditions. This may be evidence of different recommendation domains for plant density within the region. Therefore, scaling similar recommendations for fields with similar agroecological conditions can facilitate effective adoption of optimum plant densities. The objectives of this study were to: (1) determine the extent to which environmental and management practices affect optimum plant density, and (2) identify the most appropriate recommendation domain for making decisions on plant density.

## Materials and methods

To capture variability in optimum plant density of CST sweet corn, on-farm experiments were conducted in collaboration with vegetable processors in the Upper Midwest. Fields were in areas of high strategic importance within the states of Illinois, Minnesota and Wisconsin across a 5-year period. For complete details of the field experiment, see [15]. In brief, a total of thirty fields under contract with Del Monte Foods, Inc. were included. Each experiment was arranged as a randomized complete block design with two replicates. Ten levels of plant density were tested, ranging from 42,000 plants ha^-1^ to 109,000 plants ha^-1^. Green ear mass yield and the corresponding gross profit margin ($ ha^-1^) were calculated for each plant density level, and the plant density that would return maximum gross profit margin was considered the optimum plant density [15].

All experiments were nested with growers’ fields and managed by growers using their standard practices, including irrigation, fertilization, and pest management. Therefore, crop responses in this research reflect contemporary production of sweet corn grown for processing throughout the Upper Midwest.

### Environmental and management variables

Based on previous literature on plant density associations with environmental and crop management variables, twelve variables were studied. Environmental variability was accounted by climatic, edaphic, and topographic variability. Climatic variability was characterized using growing degree days (GDD) and precipitation across the growing season. Daily precipitation, minimum air temperature, and maximum air temperature were obtained from the Midwestern Regional Climate Center [24] using the nearest active weather station for each site. The GDDs were calculated using daily minimum and maximum air temperature and a base temperature of 10°C. Further, GDDs were determined from planting to tassel (GDD_pt_) and from tassel to harvest (GDD_th_). Edaphic factors included soil texture and percent organic matter. Soil samples were collected at harvest using a soil probe. A composite soil sample for each field was composed of at least six cores with core diameter 2 cm and core depth 15 cm. Soil samples were characterized for chemical (pH, micro and macro nutrient availability) and physical (particle size distribution) attributes (A&L Great Lakes Laboratories, Fort Wayne, IN). Topographic variability was accounted by latitude and longitude of the centroid of each field. Crop management variables included planting date, harvest date, and days between planting and harvest (hereafter called ‘crop duration’). Dates were expressed as day of year.

### Exploratory factor analysis and Pearson’s partial correlation analysis

Exploratory factor analysis, a commonly used multivariate technique for dimension reduction [25], was used to study covariance relationships among environmental and crop management variables. Since variables were on different scales, and to prevent variables with high variances from skewing the analysis, a correlation matrix was used for exploratory factor analysis. Exploratory factor analysis was performed using *factanal* package in RStudio [26] with varimax rotation for extracting orthogonal factor loadings. Orthogonal factor loadings are helpful as they can be interpreted similar to correlation coefficeints. Factors with eigenvalues greater than 1 were retained out of the twelve potential factors [27]. Retained factors (i.e., latent variables) represent underlying, unobservable factors. Factor scores were extracted using the *psych* package in RStudio [28]. Factor scores are the linear combinations of factor loadings and set of original variables that retain most of the variability.

Pearson’s partial correlation analysis was conducted on factor scores and optimum plant density of fields. The goal was to identify strength of associations between latent variables and optimum plant densities. All tests were declared significant at α = 0.05.

### Criteria for construction of recommendation domains

Recommendation domains can be a useful tool when choosing a target plant density for an individual field when among-field variability in optimal plant density is large [17]. The idea is to group fairly homogenous fields together that benefit from a common recommendation. There are many criterion of grouping fields, hence, numerous potential recommendation domains.

**Fig 1:**
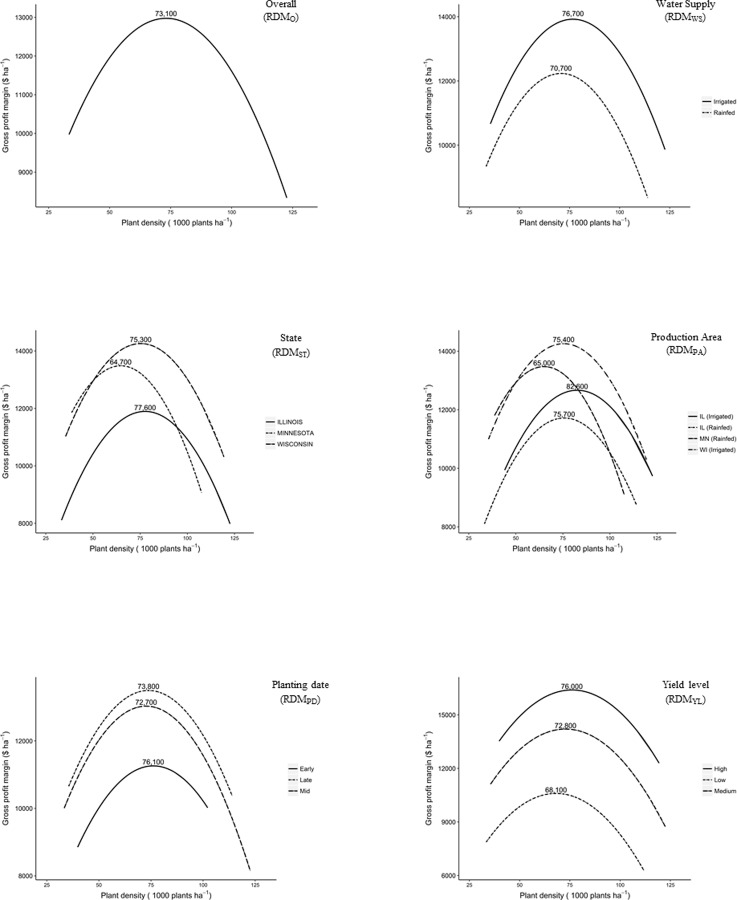
Linear mixed effects model for relationship between gross profit margin ($ ha^-1^) and plant density (plants ha^-1^) under six candidate recommendation domain models (RDM). The peak of each curve identifies the optimum plant density of each RDM level.

Based on data available for site characterization, six candidate recommendation domains models (RDM) were developed and tested (Fig 1). Candidate RDMs included ‘Overall’, ‘Water Supply’, ‘State’, ‘Production Area’, ‘Planting Date’ and ‘Yield Level’ (Fig 1). With the Overall RDM (RDM_O_), all fields were grouped into a single recommendation domain. In essence, the RDM_O_ uses a single plant density recommendation for the entire Upper Midwest. With Water Supply (RDM_WS_), fields were grouped by water supply; specifically, irrigated (N = 14) and rainfed (N = 16). The RDM_WS_ recognizes sweet corn grown under rainfed conditions may have a different optimal density than irrigated sweet corn. State (RDM_ST_) grouped fields by state; specifically, Illinois (N = 14), Minnesota (N = 5) and Wisconsin (N = 11). The RDM_ST_ attempts to account for potential differences in growing conditions and management that may exist among the three primary states in which sweet corn is grown for processing in the Midwest. Under Production Area (RDM_PA_), both state and water supply were considered; therefore, fields were grouped into Illinois-irrigated (N = 3), Illinois-rainfed (N = 11), Minnesota-rainfed (N = 5) and Wisconsin-irrigated (N = 11). The RDM_PA_ also differentiates fields by the local factory that will process sweet corn grown in the vicinity. Sweet corn planting in the Upper Midwest commences the first week of April and continues into the first week of July. For Planting Date (RDM_PD_), fields were grouped as ‘early’ if planted on or before April 30 (N = 3), ‘mid’ if planted between May 1 and June 10 (N = 19), and those planted after June 10 were considered ‘late’ planted (N = 8). Finally, in Yield Level (RDM_YL_), fields were grouped according to yield. Cluster analysis (S1 Fig) was used to group fields with similar yields together, resulting in three categories: low-yielding (N = 12), medium-yielding (N = 14) and high-yielding fields (N = 4).

### Identification of the best recommendation domain

Earlier study modelled gross profit margin response to plant density to identify the optimum plant density that would maximize gross profit margin for individual fields [15]. The same fields were classified under different recommendation domains and linear mixed effects models were fit to predict maximum gross profit margin under each candidate recommendation domain. Each model was a second order polynomial mixed effects model with domain level random intercept and slope structure, and plant density as a fixed effect. Best linear unbiased predictors (BLUPs) were extracted from each model and were used to identify the maximum gross profit margin for different levels in each candidate recommendation domain. Then, plant density corresponding to maximum gross profit margin was considered optimum plant density for the respective domain level. Grower returns also were calculated corresponding to optimum plant density for each domain level using the linear mixed effects model coefficients that were established in previous study by Dhaliwal and Williams [15].

**Fig 2:**
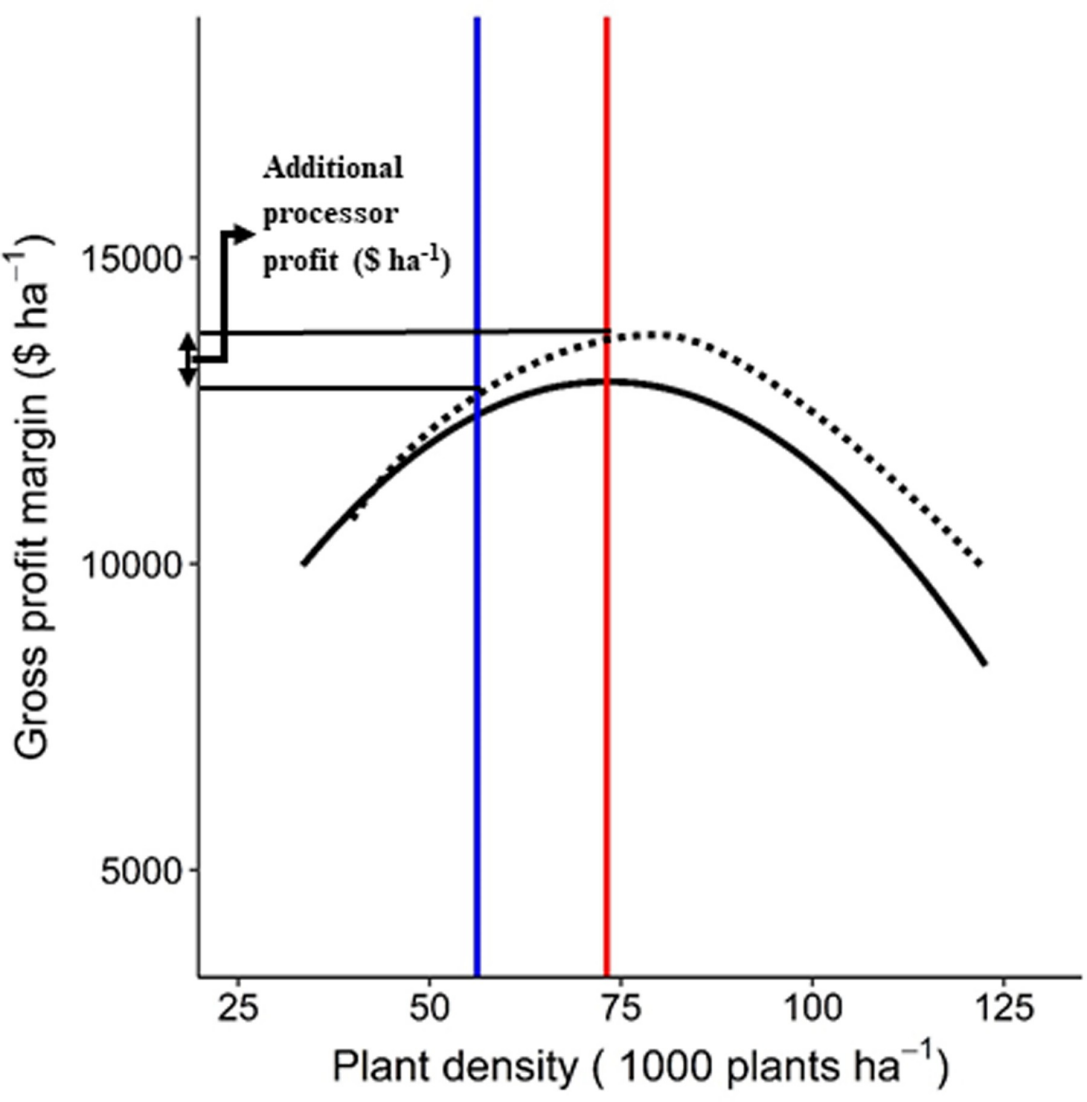
Calculation of additional processor profit ($ ha^-1^) for a field in a given level of a recommendation domain model (RDM). Red line represents the optimum plant density (plants ha^-1^) for maximum gross profit margin ($ ha^-1^) under a level of a RDM (solid black curve). Blue line represents current plant density for an individual field (dotted black curve). The difference in gross profit margin observed at the optimum plant density under RDM level and current plant density of a field give additional processor profit from the field.

The difference between gross profit margin observed at the current plant density for the field and the RDM level was identified as additional processor profit (Fig 2). Similarly, additional grower returns were calculated as difference between grower returns at RDM level optimum plant density and the field’s current plant density. Additional processor profit and grower returns were then averaged for each RDM level to calculate mean RDM values. It is noteworthy that vegetable processors decide the target plant density for processing sweet corn and their profitability is given by gross profit margins, hence, the RDM that maximized processor profits was declared the best practical choice for making decisions on plant density in CST sweet corn. The Kolmogorov-Smirnov test was used to identify differences (α = 0.05) in additional processor profitability and grower returns between RDMs [29].

## Results and discussion

Across the thirty sweet corn fields tested in this research, optimum plant density ranged from 60,850 plants ha^-1^ to 90,900 plants ha^-1^, corresponding to a maximum gross profit margin ranging from $9,000 ha^-1^ to $18,250 ha^-1^ (Table 1). Previously, Williams [13] reported CST tolerant processing sweet corn is under-planted at an average plant density of 56,000 plants ha^-1^ in the Upper Midwest. Dhaliwal and Williams [15] demonstrated shifting from current to optimum plant densities for CST processing sweet corn increased profitability of both the processor and contract grower up to $700 ha^-1^ and $105 ha^-1^, respectively, without negatively affecting ear traits important to processing.

**Table 1:** Brief description of the thirty fields in which optimum plant density for processing sweet corn was quantified in field trials in Illinois (IL), Minnesota (MN), and Wisconsin (WI) from 2013 to 2017.

Year	State	County	UTM coordinates		Soil type	Water supply	Planting date	Harvest date	Optimum plant density* (plants ha^-1^)	Maximum gross profit margin* ($ ha^-1^)
			Northing	Easting						
2013	IL	LaSalle	4604492	327342	Silt loam	Rainfed	19-Jun	6-Sep	80,850	11,750
2014	IL	Champaign	4437685	396723	Silt loam	Rainfed	27-May	11-Aug	86,100	13,280
2014	IL	Champaign	4437009	394020	Silt loam	Rainfed	27-May	13-Aug	70,350	13,210
2014	IL	DeKalb	4658787	338226	Silt loam	Rainfed	6-Jun	29-Aug	66,100	9,820
2014	IL	DeKalb	4658145	337699	Silt loam	Rainfed	6-Jun	29-Aug	69,400	11,570
2014	IL	LaSalle	4580403	335086	Silt loam	Rainfed	14-Jun	5-Sep	79,500	15,140
2014	WI	Portage	4918523	286288	Loamy sand	Irrigated	19-Jun	18-Sep	70,450	10,480
2014	WI	Portage	4916987	294885	Muck sand	Irrigated	5-Jun	9-Sep	68,250	12,220
2014	WI	Portage	4903600	284257	Loamy sand	Irrigated	23-May	25-Aug	80,550	14,350
2015	IL	Champaign	4437685	396711	Silt loam	Rainfed	22-May	5-Aug	76,200	11,360
2015	IL	Champaign	4436816	393961	Silt loam	Rainfed	22-May	6-Aug	63,450	9,890
2015	IL	Mason	4464816	249609	Sandy loam	Irrigated	29-Apr	20-Jul	72,600	10,140
2015	MN	Brown	4916794	351529	Clay loam	Rainfed	10-Jun	4-Sep	71,800	14,520
2015	MN	Redwood	4915165	333836	Clay loam	Rainfed	10-Jun	4-Sep	70,100	13,480
2015	WI	Portage	4917843	295733	Loamy sand	Irrigated	2-Jun	3-Sep	75,150	11,720
2015	WI	Portage	4904819	301859	Loamy sand	Irrigated	13-May	20-Aug	69,800	15,780
2015	WI	Waushara	4899377	292876	Loamy sand	Irrigated	16-Jun	15-Sep	66,200	16,130
2016	IL	Champaign	4437685	396720	Silt loam	Rainfed	16-May	1-Aug	70,700	13,610
2016	IL	Champaign	4436824	394114	Silt loam	Rainfed	16-May	1-Aug	74,200	10,910
2016	IL	Mason	4470084	253055	Sandy loam	Irrigated	20-Apr	22-Jul	86,950	14,320
2016	MN	Brown	4919022	328881	Clay loam	Rainfed	13-Jun	31-Aug	61,250	9,050
2016	WI	Adams	4897998	289741	Loamy sand	Irrigated	1-Jun	23-Aug	67,400	12,100
2016	WI	Portage	4920895	296506	Muck sand	Irrigated	8-Jun	6-Sep	70,100	15,380
2016	WI	Portage	4915550	290239	Loamy sand	Irrigated	19-Jun	14-Sep	90,900	18,250
2017	IL	Champaign	4437888	395145	Silt loam	Rainfed	24-Apr	28-Jul	66,050	9,510
2017	IL	Champaign	4437027	394127	Silt loam	Irrigated	16-May	7-Aug	78,350	13,630
2017	MN	Brown	4919644	340123	Clay loam	Rainfed	10-Jun	7-Sep	65,400	15,270
2017	MN	Brown	4916882	340485	Clay loam	Rainfed	11-Jun	7-Sep	60,850	15,320
2017	WI	Portage	4899176	296548	Sand	Irrigated	30-May	31-Aug	72,850	17,640
2017	WI	Portage	4920094	290860	Loamy sand	Irrigated	23-Jun	26-Sep	83,800	14,590

* Optimum plant density and maximum gross profit margin adapted from Dhaliwal and Williams, 2019

### Environment and management

Fields varied in crop management and environmental conditions. Planting dates ranged from April 24 to June 19. As such, harvest dates ranged from July 20 to September 26 (Table 1). Total crop duration ranged from 76 to 100 days (Table 2). Heat units accumulated during vegetative and reproductive growth (i.e., GDD_pt_ and GDD_th_) also varied. Soil texture varied from clay loam to silty loam to sand. Soils greater than 50 percent sand were sprinkler irrigated, whereas other soils were rainfed. Fields received variable precipitation, ranging from 20.3 cm to 59.5 cm from planting to harvest (Table 2). Fields used in this research represent the wide range of conditions in which processing sweet corn is grown in the Upper Midwest.

**Table 2:** Summary statistics of the environmental and crop management variables of thirty fields in which optimum plant density for processing sweet corn was quantified in field trials in Illinois, Minnesota, and Wisconsin from 2013 to 2017. Universal Transverse Mercator (UTM) uses a 2-dimensional Cartesian coordinate system to give locations on the surface of the Earth. GDDpt and GDDth represent growing degree days observed during planting-tassel and tassel-harvest, respectively.

Variable	Units	Mean	Standard deviation	Minimum	Maximum
Latitude	UTM	4717924	219871	4436816	4920895
Longitude	UTM	330573	46532	249609	396723
Planting date	day of year	150.7	16.7	111	174
Harvest date	day of year	236.8	18.1	201	269
Crop duration	days	87.1	7.0	76	100
Organic matter	%	4.5	3.1	0.7	16.8
Sand	%	44.6	36.2	5	94
Silt	%	36.4	26.4	1	71
Clay	%	19	11.8	4	38
Precipitation	cm	37	10.3	20.3	59.5
GDD_pt_	heat units	1,070	83.6	825	1,179
GDD_th_	heat units	615.3	93.7	452	852

Several environmental and crop management variables were correlated. Latitude was positively correlated with planting date (ρ = 0.64), harvest date (ρ = 0.82), and crop duration (ρ = 0.62; Table 3). Late planting dates are observed at higher latitudes pertaining to the environmental conditions, especially air temperature and soil conditions. Sweet corn growers have found that cold wet soils lead to slow germination in shrunken-2 (*sh-2*) sweet corn. Hassell et al. [30] reported *sh-2* type sweet corn hybrids require slightly higher temperatures for germination than sugar enhanced (*se*) and sugary (*su*) sweet corn. They found *sh-2* type sweet corn hybrids took minimum time for germination at air temperature around 22°C [30]. Long et al. [31] also reported planting date for field corn increased from 60^th^ to 100^th^ day of year as latitude increased from 25°N to 35°N.

**Table 3:** Pearson’s partial correlation coefficients between environmental and crop management variables of thirty fields in which optimum plant density for processing sweet corn was quantified in field trials in Illinois, Minnesota, and Wisconsin from 2013 to 2017. Coefficients in bold are significant at α = 0.05. GDDpt and GDDth represent growing degree days observed during planting-tassel and tassel-harvest, respectively.

	Latitude	Longitude	Planting date	Harvest date	Crop duration	Organic matter	Sand	Silt	Clay	Precipitation	GDDpt
**Latitude**	1.00										
**Longitude**	**-0.60**	1.00									
**Planting date**	**0.64**	-0.16	1.00								
**Harvest date**	**0.82**	**-0.38**	**0.92**	1.00							
**Crop duration**	**0.62**	**-0.62**	0.01	**0.39**	1.00						
**Organic matter**	0.06	0.25	0.21	0.11	-0.22	1.00					
**Sand**	**0.71**	**-0.86**	0.18	**0.44**	**0.72**	-0.21	1.00				
**Silt**	**-0.80**	**0.89**	-0.26	**-0.52**	**-0.72**	0.16	**-0.98**	1.00			
**Clay**	**-0.40**	**0.64**	0.05	-0.18	**-0.59**	0.30	**-0.88**	**0.75**	1.00		
**Precipitation**	-0.12	0.07	-0.23	-0.30	-0.21	0.16	-0.16	0.10	0.26	1.00	
**GDDpt**	0.18	0.27	**0.55**	**0.44**	-0.17	0.30	-0.17	0.10	0.28	-0.29	1.00
**GDDth**	**-0.60**	0.30	**-0.75**	**-0.72**	-0.07	-0.02	-0.35	**0.40**	0.18	0.02	**-0.41**

As expected, edaphic factors including sand, silt and clay variables were highly correlated with each other (ρ = -0.98 to 0.75). Likewise, GDD_pt_ was positively correlated to planting date (ρ = 0.55) and, GDD_th_ was negatively correlated to planting date (ρ = -0.75) and harvesting date (ρ = -0.72).

Exploratory factor analysis identified underlying common factors explaining most of the variation in environmental and crop management variables. Two factors were retained and, collectively, accounted for 62.6 percent of the total variance (Table 4). Factor 1 had positive loadings for planting date, harvest date, latitude, and GDD_pt_, whereas GDD_th_ had a negative loading in factor 1. Factor 1 was interpreted as the ‘growing period’ factor. Longitude, sand, and clay loaded positively into factor 2 (Table 4). Factor 2 was interpreted as the ‘soil type’ factor. Communality values were high for most of the variables (h^2^ = 0.57 to 0.99), indicating the factor analysis model satisfactorily explained total variability contributed by individual environmental and crop management variables. Kaspar et al. [32] reported the factor comprised of high positive loadings from silt, clay and negative loadings from sand, slope and soil color, were positively associated with field corn yield in dry growing seasons of central Iowa. However, the same factor was negatively associated with field corn grain yields in wet growing seasons. Such outcomes were determined to be the result of soil physical properties favoring soil water retention, which was beneficial to the crop in dry years, but damaging in wet years due to extended periods of saturated soils [32].

**Table 4:** Exploratory factor analysis results, based on varimax rotation, using the correlation matrix of environmental and crop management variables from thirty fields in which optimum plant density for processing sweet corn was quantified in field trials in Illinois, Minnesota, and Wisconsin from 2013 to 2017. Factor loadings from variables that were greater than 0.400 in magnitude are in bold.

Variable	Factor_1_	Factor_2_	Communality (h^2^)
	^a^Orthogonally rotated loadings		
Latitude	**0.675**	**-0.648**	0.88
Longitude	-0.146	**0.883**	0.81
Planting date	**0.964**		0.95
Harvesting date	**0.932**	-0.293	0.97
Organic matter	0.227	0.225	0.12
Sand	0.136	**-0.968**	0.99
Clay	0.149	**0.810**	0.99
Precipitation	-0.207		0.15
GDD_pt_	**0.562**	0.268	0.39
GDD_th_	**-0.699**	0.225	0.57
Eigen values	3.22	3.05	
Total variance (%)	32.1	30.5	**62.6**
Common variance (%)	51.3	48.7	**100**

^a^varimax rotation.

Despite the logical outcome of factor analysis, neither ‘growing period’ or ‘soil type’ factors were main drivers of variability in optimum plant density. Pearson’s partial correlation coefficients of ‘growing period’ and ‘soil type’ with optimum plant density were low (*ρ*_*1*_ = -0.14 and *ρ*_*2*_ = -0.09, respectively) and non-significant (P = 0.47 and 0.65, respectively). Apparently, there were other unmeasured variables responsible for varied optimum plant densities. A common limitation encountered with on-farm studies is the limited access to the growers’ farms, thus setting a trade-off between the quality and quantity of data accessed from those farms [33]. Moreover, multivariate techniques like exploratory factor analysis perform best when the number of observations exceeds the number of variables by one order of magnitude [34, 35].

### Recommendation domains

Optimum plant density under RDM_O_ was 73,100 plants ha^-1^ (Fig 1). The average current plant density is 56,000 plants ha^-1^ [13,15]. Increasing plant density from current to the level determined by RDM_O_, vegetable processors and contract growers may realize a profit increase averaging $430 ha^-1^ and $81 ha^-1^ (Table 5). Recommended plant density for CST sweet corn under RDM_O_ is higher than the previously reported optimum plant densities for sweet corn in the Upper Midwest [9, 36].

**Table 5:** Mean additional processor profit ($ha^-1^) and grower returns ($ ha^-1^), standard error, and sample size for each level of the six candidate recommendation domain models (RDM). RDM mean additional processor profit and grower returns were determined using the weighted average of RDM levels. For a description of how additional processor profit were calculated, see Fig 2.

Recommendation domain model (RDM)	RDM level and mean	Sample size	Additional processor profit	Standard error	Additional grower returns	Standard error
			$ ha^-1^			
**Overall**	**RDM**_**O**_ **mean**	**30**	**430**	**72**	**81**	**10**
**Water supply**	Irrigated	14	524	113	94	12
	Rainfed	16	370	89	72	13
	**RDM**_**WS**_ **mean**	**30**	**442**	**77**	**82**	**11**
**State**	Illinois	14	443	132	75	78
	Minnesota	5	266	107	62	15
	Wisconsin	11	509	133	97	14
	**RDM**_**ST**_ **mean**	**30**	**438**	**58**	**81**	**9**
**Production-area**	IL-Irrigated	3	600	180	76	5
	IL-Rainfed	11	429	146	76	24
	MN-Rainfed	5	268	110	63	15
	WI-Irrigated	11	509	134	98	14
	**RDM**_**PA**_ **mean**	**30**	**448**	**55**	**82**	**7**
**Planting date**	Early	3	290	189	39	31
	Mid	19	437	71	81	11
	Late	8	475	223	97	23
	**RDM**_**PD**_ **mean**	**30**	**432**	**36**	**81**	**11**
**Yield level**	Low	12	336	66	54	11
	Medium	14	451	106	90	12
	High	4	737	255	126	34
	**RDM**_**YL**_ **mean**	**30**	**443**	**90**	**81**	**17**

Optimum plant density under RDM_WS_ for irrigated and rainfed fields was 76,000 and 70,700 plants ha^-1^, respectively (Fig 1). Using the plant density recommendations under RDM_WS_, growers may realize additional $72 ha^-1^ and $94 ha^-1^ in rainfed and irrigated fields in the Upper Midwest (Table 5). Under RDM_WS_, irrigated fields showed $155 ha^-1^ more in processor profits than fields under rainfed conditions (Table 5). Recommended plant densities under RDM_WS_ agree with the findings of previous studies that report fully irrigated production systems can sustain higher plant densities compared to rainfed systems [9, 36]. Piana et al. [37] reported 107,000 plants ha^-1^ was optimum plant density for field corn under irrigated conditions. Similarly, Silva et al. [38] and Takasu et al. [39] reported optimum plant density for maximum grain yield in irrigated field corn were 100,000 plants ha^-1^ and 90,000 plants ha^-1^, respectively. In a Minnesota study of field corn, optimum plant densities were reduced 12 percent when rainfall exceeded long-term averages by approximately 50 percent during the growing season [40]. Water becomes a limiting factor for biomass production in field corn at higher plant densities under rainfed conditions [41].

Under RDM_ST_, optimum plant densities for fields in Illinois, Minnesota and Wisconsin were 77,600 plants ha^-1^, 64,700 plants ha^-1^ and 75,300 plants ha^-1^, respectively (Fig.1). Based on RDM_ST_, plant density recommendations were more profitable for processors in Illinois ($443 ha^-1^) and Wisconsin ($509 ha^-1^) than Minnesota ($266 ha^-1^) (Table 5). These results were consistent with Stanger and Lauer [23] and Coulter [42] who reported economic optimum plant densities for field corn were similar for Wisconsin (83,000 plants ha^-1^) and Illinois (79,800 plants ha^-1^). In contrast, Van Roekel and Coulter [43] reported plant densities in range of 81,700 to 107,900 plants ha^-1^ maximized grain yields in field corn in the southern Minnesota. Maximum gains in grower returns were observed in Wisconsin ($97 ha^-1^) at plant density recommendations under RDM_ST_.

Under RDM_PA_ fields were grouped based on both water supply and state. Optimum plant densities under RDM_PA_ ranged from 65,000 to 82,600 plants ha^-1^ (Fig 1). Based on recommendations from RDM_PA_, vegetable processors may realize additional profits ranging from $268 ha^-1^ to $600 ha^-1^. Optimum plant density in field corn differs among latitude zones in the United States [5]. Three decades ago, field corn grain yield in Illinois was maximized at 56,300 plants ha^-1^ to 76,750 plants ha^-1^. In the present work, Minnesota-rainfed processor profit was $268 ha^-1^ and grower returns were $63 ha^-1^ by following plant density recommendations under RDM_PA_.

The RDM_PD_ identified optimum plant densities for fields grouped by three planting date windows (Fig 1). Under RDM_PD_, early-planted fields (76,100 plants ha^-1^) had higher optimum plant densities than mid- (72,700 plants ha^-1^) and late-planted fields (73,800 plants ha^-1^). Williams [44] reported late-June planted sweet corn had lower yields than early-May planted sweet corn due to lower water supply and increased disease incidence in late-June plantings. Williams [44] also found early-July planted sweet corn took 23 to 35 percent fewer days from crop emergence to silking period, however, mid-June and early-July plantings also resulted in plants with fewer leaves and slower rates of leaf appearance. Nielsen et al. [45] reported GDDs accumulated from planting to silk emergence were 34 units less for June plantings than early May plantings in dent corn (*Zea mays* L.var. *indentata*). Similarly, [46] recorded higher grain yields in early-April plantings compared to late-May plantings for field corn. Conceivably, using higher plant densities for early planting dates would allow the crop to take advantage of favorable growing conditions which include more days of available solar radiation, potentially avoid some diseases, and risk of late-season drought. Currently, vegetable processors reduce plant densities 5–10 percent for the latest planting dates (C. Bahr, personal communication).

Under RDM_YL_, optimum plant densities for low-, medium-, and high-yielding fields were identified. The RDM_YL_ showed optimum plant density for low-yielding fields (68,100 plants ha^-1^) was lower than medium-yielding (72,800 plants ha^-1^) and high-yielding fields (76,000 plants ha^-1^). These results show a similar trend as field corn, as evidenced by low-yielding environments (less than 7 Mt ha^-1^) were limited to 73,000 plants ha^-1^ whereas high-yielding environments (greater than 13 Mt ha^-1^) required at least 100,000 plants ha^-1^ [5]. Plant density recommendations under RDM_YL_ resulted in the maximum additional processor profit ($737 ha^-1^) and grower returns ($126 ha^-1^) in the high-yielding fields (Table 5). Contrarily, low-yielding fields showed the least gains in processor profits and gross returns among all three yield levels.

Gains in processor profit or grower returns were the differences between gross profit margin or gross returns observed at the current plant density for the field and the RDM level. The RDM mean additional processor profit and grower returns is the average value across all of the RDM’s levels. Kolmogorov-Smirnov tests showed that mean additional processor profit and grower returns were statistically similar across RDMs. Nonetheless, for the vegetable crop industry to benefit from increasing plant density of CST hybrids, they need research-based guidance on determining plant density, and practical differences exist among RDMs.

Plant density recommendations under RDM_PA_ resulted in the maximum gain in processor profits ($448 ha^-1^) and grower returns ($82 ha^-1^), as well making it the most suitable RDM for deciding plant densities for fields across the Upper Midwest. Also, RDM_PA_ reduced the variability for additional processor profit and grower returns within each level (i.e., production area) as shown by smaller standard deviations relative to other RDMs (Table 5). Plant density recommendations based on RDM_PA_ make the most of genetic potential of CST processing sweet corn hybrids. Also, RDM_PA_ can be viewed as an improved version of RDM_WS_ and RDM_ST_ as it accounts for both water supply and state factors. Moreover, adopting recommendations for optimum plant density from RDM_PA_ would be quite feasible. The four levels of RDM_PA_ are already distinct within the vegetable processing industry. Typically, one or more processing plants exist within each state. Contract sweet corn production is managed by field supervisors assigned to the four levels of RDM_PA_. Those field supervisors make decisions for their contract fields within their assigned territory, including plant density. Therefore, plant density recommendations based on RDM_PA_ are most likely to lead to successful adoption across fields in the Upper Midwest to realize increased profitability to both processors and their contract growers.

## Conclusion

Variability in optimum plant density for CST sweet corn exists in fields across the Upper Midwest; however, a research-based approach to guide plant density recommendations is lacking. To maximize profitability from using increased plant densities of CST sweet corn, processors should decide plant densities tailored to the local growing conditions. Of six different recommendation domains tested, plant density recommendations under RDM_PA_ maximized gains in processor profits ($448 ha^-1^) and grower returns ($82 ha^-1^). Moreover, RDM_PA_ groups fields into a structure the sweet processing industry already utilizes for field-level decision making.

## Supporting information

S1 Fig K-means clustering results on yield components for all fields. Yield components included case production (cases ha^-1^), ear number per plant, ear mass per plant (kg plant^-1^), green ear mass (Mt ha^-1^), and gross profit margin ($ ha^-1^) of individual fields.
(PDF)

S1 File Raw data used for all analyses in the manuscript. (CSV)
