# Supplementary figures and images for: Understanding variability in optimum plant density and recommendation domains for crowding stress tolerant processing sweet corn
Source: PLoS One. 2020 Feb 7;15(2):e0228809. doi: 10.1371/journal.pone.0228809 (PMC7006923; doi:10.1371/journal.pone.0228809)

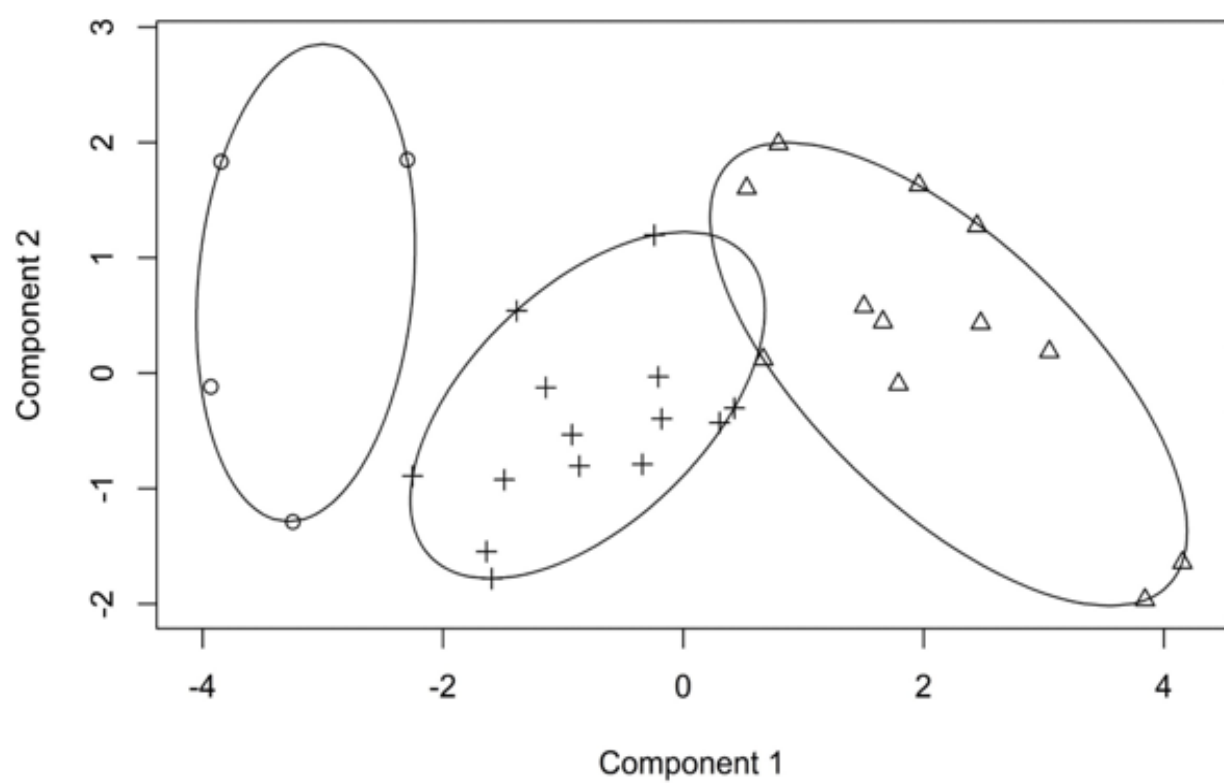

+ Low yielding    △ Medium yielding    ○ High yielding

Supplement: S1 Fig — Yield components included case production (cases ha-1), ear number per plant, ear mass per plant (kg plant-1), green ear mass (Mt ha-1), and gross profit margin ($ ha-1) of individual fields. (PDF) [file pone.0228809.s001.pdf]
